# The Million Death Study in India: Can It Help in Monitoring the Millennium Development Goals?

**DOI:** 10.1371/journal.pmed.0030103

**Published:** 2006-02-14

**Authors:** Zulfiqar A Bhutta

## Abstract

Bhutta argues that the investigators of a new demographic survey, the Million Death Study in India, must ensure that the survey is relevant to the Millenium Development Goal targets that India has set itself.

The Registrar General of India's Sample Registration System (SRS), a large routine demographic survey, is the country's primary system for the collection of fertility and mortality data ( Box 1). It is widely regarded as an innovative way of capturing essential information on mortality patterns that may influence public health policy. However, although this monitoring system has been in place since 1971, there are relatively few evaluations to indicate if the SRS is truly representative or functional as a robust monitoring system.

The Million Death Study in India is an ambitious project being undertaken by the SRS in close collaboration with the Centre for Global Health Research at the University of Toronto, leading Indian and other overseas academic institutions, and the Indian Council of Medical Research. The study will use the SRS framework to obtain information, from a reasonably large cohort of deaths (1 million deaths over 16 years), on the underlying causes of child and adult deaths, as well as key risk factors for these deaths (behavioral, physical, environmental, and, possibly, genetic). In a new study in *PLoS Medicine*, Prabhat Jha and colleagues report on the rationale, design, and implementation of the Million Death Study in India [ 1].

The study will use a combination of methods, looking at both a retrospective and a prospective cohort of deaths. These methods include case control studies, risk assessments, and genetic association studies. Given the unique characteristics of the health-care system in India, and the country's enormous double burden of infectious and noncommunicable diseases, the Million Death Study will provide valuable information for India that may also be relevant to other developing countries.

The researchers involved in the study have shown great foresight in making their protocols available for public scrutiny. It may, therefore, be worthwhile to ask a few pertinent questions at this early stage in the project.

## Is the “Large Sample” Truly Representative?

Is the SRS framework adequate for India's current and future surveillance needs? While the scale of the study and the sample of 1 million deaths spanning the period 1998–2014 is daunting, one can see some limitations of this unique exercise given the population size and diversity in India. The study is largely embedded within the revised SRS framework, and capitalizes on the existing system. Although the new SRS sample frame is larger than before, the 7,597 sampling units cover only 7.6 million people out of India's population of just over 1 billion people. The new sample covers only half a million of an estimated annual 9.5 million deaths in India.

Public health surveillance and monitoring systems have a key role in local health policymaking, so it would have been valuable for the SRS to have given information with district-level specificity. It is also unclear if the SRS sample covers pockets of deprivation in urban and periurban slums, where health care and health indicators are even worse than in rural populations [ 2]. Given the increasingly devolved health-care system, any surveillance and monitoring system must have local specificity, and given the scope and timescale of the study, these issues are best addressed at the outset. This study could have been the stimulus to expand the SRS sample size to reflect district level trends, as well as maternal mortality (a reduction in maternal mortality is an important Millennium Development Goal [MDG]).

The major limitation in terms of expanding the SRS is cost. The current study is being undertaken with an incremental budget of a mere US$2 million. While the resources are clearly a limitation for this study, the low level of public health spending in the region nevertheless largely reflects misplaced priorities [ 3]. I would argue that perhaps the study itself might have been the impetus to expand the SRS sample to a more representative sample with the power to track important MDG indicators in all strata.

## Is the Focus Appropriate?

The Million Death Study largely focuses on adult mortality and, as indicated by the authors, it may not have sufficient power to address maternal mortality. This is unfortunate because the persistently high burden of maternal morbidity and mortality is a unique challenge for South Asia [ 4]. While the constraints of an adequate population sample for maternal mortality are understandable, it would have been useful to see a few more reproductive health indicators in the current repertoire of risk factors that the investigators intend to track. With the global health community now focusing on achieving the MDGs [ 5], it is important to critically review and expand the repertoire of studies on maternal and childhood mortality. India has a high burden of fetal malnutrition [ 6], so it is also important to strengthen the emerging evidence of the link between maternal and fetal malnutrition, childhood growth, and long-term adverse outcomes such as diabetes and premature cardiovascular disease [ 7–8]. It may be possible to include variables on size at birth and early growth parameters within the planned substudies of the Million Death Study, as these variables may be important correlates of adult-onset diseases and premature mortality.

## Are the Tools Robust?

Although the validity of routinely monitoring health data by health-system employees can be questioned, the proposed study will have additional components that will involve secondary analyses of the data by external staff. It would also be important to have protocols in place for regular external validation of the primary data. Previous evaluations have shown close concordance between the data on smoking and alcohol consumption generated by the Special Fertility and Mortality Survey (the baseline survey of the Million Death Study) [ 9] and the data from the National Family Health Survey 2 (the National Family Health Survey is a large-scale, multiround survey coordinated by India's International Institute for Population Sciences) [ 10], suggesting that the SRS data are indeed robust. Nevertheless, such cross-checking of the primary SRS data would be necessary through additional periodic surveys.

The challenge is to ensure that the study is relevant to the MDG targets that India has set itself.

The Million Death Study proposes to use verbal autopsy instruments, for example, recording details of deaths as reported by family or friends to a trained but nonmedical fieldworker. Verbal autopsy instruments are already in use for recording SRS deaths, and they have been validated for adult mortality, except for those at the extremes of age. The infant and child verbal autopsy tool [ 11] referred to in the proposed study has not been validated for use within the neonatal period, and several modifications have been made to adapt it to such use [ 12–14]. Given that almost 50% of all infant deaths in India occur in the neonatal period [ 15], the validation of the verbal autopsy tool employed in the SRS is critically important. Such studies for validation of modified neonatal verbal autopsy instruments are currently underway (S. Qazi, World Health Organization, personal communication).

While the current study plans to focus on the “usual suspects” in causing neonatal deaths, such as deaths due to birth asphyxia, prematurity, and serious infections, it is equally important to be aware of emerging issues in neonatal health. The high global burden of stillbirths, especially those that occur in the intrapartum period, has been recently recognized [ 16]. Given that these deaths may represent the hidden burden of birth asphyxia and perinatal care, it would be appropriate to evaluate the importance of fresh stillbirths in the current framework (the Million Death Study framework appears to exclude stillbirths). The same may apply to infant deaths relating to malformations such as neural tube defects [ 17], which may represent both widespread nutritional deficiencies and genetic polymorphisms. The planned study offers a unique opportunity to capture the true burden of premature mortality and potentially decrease handicaps relating to perinatal events and congenital malformations, on which almost no data exist from large population settings.

## Ethical Considerations

The planned inclusion of genetic studies and the setting up of bio-banks (collections of samples of bodily substances that are, or can be, associated with personal data) are a welcome initiative, as these studies have important implications for public health preventive strategies [ 18]. However, the use of such tools and research must be strictly regulated by ethical guidelines and criteria, such as those developed by the Indian Council for Medical Research [ 19]. The ethical issues involved in the use of biological materials in population surveys are a subject of intense debate, and while there are few universally applicable guidelines [ 20], the Million Death Study offers an opportunity for progress in this dynamic field. Although these ethical aspects are not fully elaborated in the current proposal, one hopes that the investigators will use the available opportunity to address them. While these ethical issues may be important in mortality studies, they are particularly germane to the planned nested case-control studies.

## Conclusion

In summary, despite the questions I have raised above, the Million Death Study is one of the most important projects in population health, with the real potential of addressing priority issues in public health. The challenge is to ensure that the study is relevant to the MDG targets that India has set itself, and that it also informs public health policy as it evolves. Addressing this challenge may mean potentially increasing the sampling frame to make the data more relevant at the district level, and including additional maternal and newborn health indicators within the current repertoire. Pushing the envelope in an already large project at this stage may yield much greater dividends in due course.

**Box 1. Sample Frames of India's SRS**

“There are two SRS sample frames. The first SRS sample frame covers 6.3 million people (including 2.9 million adults aged 25 years or older) in all 28 states and seven union territories of India. An average of 150 households are drawn from each of 6,671 sample units (4,436 rural and 2,235 urban), which in turn are selected using 1991 census data. The new SRS sample frame covers about 7.6 million people (including 3.5 million adults aged 25 years or older) in all 28 states and seven union territories of India. Households are drawn from 7,597 sample units (4,433 rural and 3,164 urban) selected from the 2001 census” [ 1].

## Abbreviations

MDG: Millennium Development Goal
SRS: Sample Registration System
